# Malaria Risk in Travelers

**DOI:** 10.3201/eid1103.040677

**Published:** 2005-03

**Authors:** Helena Hervius Askling, Jenny Nilsson, Anders Tegnell, Ragnhild Janzon, Karl Ekdahl

**Affiliations:** * Swedish Institute for Infectious Disease Control, Stockholm, Sweden; † Karolinska University Hospital, Stockholm, Sweden; ‡ Karolinska Institute, Stockholm, Sweden

**Keywords:** Malaria, travel, Sweden, risk, case-control, database, research

## Abstract

Imported malaria has been an increasing problem in several Western countries in the last 2 decades. To calculate the risk factors of age, sex, and travel destination in Swedish travelers, we used data from the routine reporting system for malaria (mixture of patients with and without adequate prophylaxis), a database on travel patterns, and in-flight or visa data on Swedish travelers of 1997 to 2003. The crude risk for travelers varied from 1 per 100,000 travelers to Central America and the Caribbean to 357 per 100,000 in central Africa. Travelers to East Africa had the highest adjusted odds ratio (OR = 341; 95% confidence intervals [CI] 134–886) for being reported with malaria, closely followed by travelers to central Africa and West Africa. Male travelers as well as children <1–6 years of age had a higher risk of being reported with malaria (OR = 1,7; 95% CI 1.3–2.3 and OR = 4,8; 95%CI 1.5–14.8) than women and other age groups.

## Abstract 2

Malaria risk around the world was assessed by using Swedish surveillance data from 1997 to 2003 with an extensive travel database as denominator.

Imported malaria has been an increasing problem in Sweden and other Western countries in the last 2 decades. Two possible reasons for this increase are the increase in the number of travelers to tropical countries, as well as a growing number of immigrants from malaria-endemic countries (*1*–*3*). Even though the number of malaria cases has been declining during the past years in Sweden (*4*), the risk for travelers is still evident and should be a concern for physicians who give pretravel advice or evaluate a returning traveler with fever.

Several studies have assessed malaria risk in travelers to specific countries (*5*–*8*). The risk of a traveler’s acquiring malaria has been considered highest in sub-Saharan Africa and Papua New Guinea, intermediate on the Indian subcontinent, and low in Southeast Asia and Latin America. The numbers assigned to the relative risk in these regions, however, are quite variable (*9*–*12*). The total number of travelers is often unknown, and most reports based on national reporting data therefore lack a denominator (*2*,*9*). Hence, making a risk assessment on the basis of such figures is difficult. Other approaches to achieve risk estimates are case-control studies. In 1 such Danish study, the country-specific risk for acquiring malaria varied from 714 per 100,000 travelers to Ghana to 2.5 per 100,000 to Thailand (*13*).

To our knowledge, no previous study based on national data over an extended period has related the number of cases of malaria diagnosed in returning travelers from malaria-endemic areas to continuously collected data on the total number of travelers to that same area. Through access to one of Europe’s largest ongoing surveys on travel patterns (*14*) and to data on reported malaria, we analyzed the risk factors for malaria in returning Swedish travelers from 1997 to 2003.

## Materials and Methods

### Cases and Controls

Cases were derived from the routine Swedish reporting system. Malaria is a reportable disease in Sweden, and all patients in whom the disease is diagnosed are reported from both the clinician treating the patient and the microbiology laboratory confirming the diagnosis. By using a unique personal identification number, issued to all Swedish residents, the 2 reporting sources can be linked. Thus, all cases reported from 1997 to 2003 were included. Since routine surveillance data were not sufficiently detailed regarding information on prophylaxis, the patients included a mixture of those with and without adequate prophylaxis. To assess travel risk specifically, newly arrived immigrants and refugees, who lack a personal identification number, were excluded from the study.

Controls were obtained from the Swedish Travel and Tourist Database, a commercial ongoing survey, based on a randomized selection of 2,000 members of the Swedish population each month (*14*). These persons are interviewed by telephone in regard to all overnight travel (business as well as pleasure) in the preceding month, and the data are weighted and extrapolated to estimate the total numbers of Swedish travelers. When the number of respondents has been too low to give a reliable estimate of the total number of travelers to a specific country, regions (rather than single countries) are given in this database. This practice was used for all malaria-endemic countries in this report, except for Thailand, where country-specific data were available for the period 2001–2003.

Out of the total database, which contained detailed information from almost 170,000 interviews, information about all respondents who had traveled to malaria-endemic areas from 1997 to 2003 was extracted. To evaluate data quality in the travel database, the weighted and extrapolated estimates of total numbers of travelers to Thailand, India, The Gambia, and South Africa were compared with in-flight or visa data on Swedish travelers. These figures were obtained by courtesy of the embassies of the respective country in Sweden, except for The Gambia, where the figures were supplied by the Central Statistics Department of the Gambia, (through the courtesy of the Swedish embassy in Dakar, Senegal). For each study participant (patients and controls), we used the following information: age, sex, year and month of infection (patients); year and month of travel (controls); country of infection (patients); and country/region of travel (controls). No data on any illness were available for controls. The latest available information on annual malaria incidence among the local population in the studied countries and regions was accessed from the World Health Organization (WHO) (*15*).

### Statistics

The risk for disease per 100,000 travelers, with 95% confidence intervals (95% CI), was calculated by using reporting data as numerator and the estimated total numbers of travelers from the travel database as denominator. For the 4 specific countries mentioned above, the risk per 100,000 travelers was also calculated by using in-flight and visa data as denominator. Since malaria is a rare disease in Sweden and controls were chosen randomly from the entire Swedish population, we could use odds ratios (OR) with corresponding 95% CI as relative risk estimates to assess the association between risk factors (age, sex, and travel destination) and outcome (being reported with malaria).

Each risk factor was first analyzed in a univariate model. To adjust for confounding, we then used a multivariate logistic regression model with these variables, and we also included month of travel or infection. The parameter with the lowest OR in each category was used as reference in the models. Likelihood ratio statistics were used to assess whether each variable in the model contributed significantly to the model and to test for interaction. All analyses were performed with Stata 6.0 software (Stata Corporation, College Station, TX, USA).

The travel database contains aggregated data only. Reportable data are regulated by the Communicable Disease Act and contain full personal identification. The subset of the reporting database abstracted for this study did not contain any information that could be linked to a specific person. The study was approved by the Ethics Committee of the Karolinska Institute, Stockholm, Sweden.

## Results

From 1997 to 2003, a total of 975 persons were reported with malaria in Sweden; 118 of them were newly arrived immigrants or refugees and thus excluded from further analysis (Table 1). Of the remaining 857 persons, 348 were infected with *Plasmodium falciparum*, 178 with *P. vivax*, 47 with *P. ovale*, and 15 with *P. malariae* (Tables 2 and 3). In 269 patients, the report did not contain data on *Plasmodium* species. Most of these patients were seen in 1997 before the full implementation of a new reporting system that year. Little variation occurred, either in the number of reported cases or the species distribution over the period, except in the last year studied (2003), which had <65% of the mean of reported cases for the preceding years. This low figure in 2003 was mainly due to a decreased number of reported *P. falciparum* cases.

**Table 1:** Reported cases of malaria in Sweden, by group of patients, 1997–2003

Category of infection	1997	1998	1999	2000	2001	2002	2003	Total
Immigrants/refugees	10	16	23	14	13	24	18	118
Travel associated	157	144	124	112	127	111	82	857
Total	167	160	147	126	140	135	100	975

**Table 2:** Reported travel-associated cases of malaria in Sweden, 1997–2003

Source of infection	1997	1998	1999	2000	2001	2002	2003	Total
*Plasmodium falciparum*	10	56	55	60	58	65	44	348
*P. malariae*	0	0	1	5	4	4	1	15
*P. ovale*	2	11	4	8	7	10	5	47
*P. vivax*	12	34	33	19	40	21	19	178
Unspecified species	133	43	31	20	18	11	13	269
Total	157	144	124	112	127	111	82	857

**Table 3:** Reported travel-associated cases of malaria in Sweden by species of malaria, 1997–2003

Region	*Plasmodium falciparum*	*P. malariae*	*P. ovale*	*P. vivax*	Unspecified	Total
Arab countries and Iran	0	0	0	2	2	4
Indian subcontinent	4	0	3	42	25	74
East Asia	16	1	0	64	30	111
West Africa	141	5	14	9	73	242
East Africa	87	5	14	31	79	216
Central Africa	58	4	9	8	28	107
Southern Africa	40	0	6	6	26	78
Central America + Caribbean	1	0	0	4	2	7
South America	1	0	1	12	4	18
Total	348	15	47	178	269	857

A total of 16,255 persons with overnight travel abroad were recorded in the travel database for the period 1997–2003. Of these, 881 (projected to a total of 3.5 million travelers) had traveled to malaria-endemic countries or regions, as defined by WHO (*16*) and were included as controls (Tables 3 and 4). Of the travel destinations, east Asia (mainly Thailand) was dominant.

**Table 4:** Estimated number of travelers to malaria-endemic areas, respondents in the tourist database (controls), and reported patients with travel-associated malaria, 1997–2003*

Age/sex/region†	Estimated no. of travelers	Controls	Reported cases	Risk per 100,000	95% CI	Multivariate odds ratio	95% CI	Incidence/ 100,000
Total	3,560,000	881	857	24	22–26	–	–	36,865
<6 y	70,000	18	38	54	31–95	4.8	1.5–14.8	No data
7–18 y	300,000	74	91	30	22–41	2.7	1.1–6.0	No data
19–45 y	1,630,000	404	506	31	27–35	4.1	1.9–9.0	No data
46–65 y	1,340,000	331	205	15	13–18	2.0	0.9–4.3	No data
>65 y	220,000	54	17	7.7	4–13	1.0	Reference	No data
Men	1,790,000	444	536	30	26–34	1.7	1.3–2.3	No data
Women	1,770,000	437	321	18	16-21	1.0	Reference	No data
Arab countries and Iran‡	220,000	44	4	1.8	0.7–5.1	1.7	0.5–6.4	1,279
Indian subcontinent	120,000	31	74	62	41–94	57.4	23–141	366
East Asia	2,050,000	517	111	5.4	4.4–6.6	5.6	2.5–12.5	205
West Africa	80,000	22	242	302	196–468	277	112–683	13,356
East Africa	90,000	18	216	240	148–388	341	134–866	7,126
Central Africa	30,000	8	107	357	174–732	317	108–930	5,508
Southern Africa	170,000	42	78	46	32–67	49.6	21–119	7,742
Central America + Caribbean	550,000	43	7	1.3	0.6–2.7	1.0	Reference	155
South America	250,000	61	18	7.2	4.3–12.2	7.1	2.7–18.4	1,205

*With a multivariate odds ratios (with 95% confidence intervals [CI]) for the risk factors age, sex, and travel destination from a logistic regression model, and incidence per 100,000 inhabitants as reported to the World Health Organization (*15*). †Malaria-free countries as defined by the World Health Organization (*16*). ‡Arab countries and Iran = Bahrain,† Iraq, Iran, Jordan,† Kuwait,† Lebanon,† Oman, Qatar,† Saudi Arabia, Syria, United Arab Emirates, Yemen; Indian Subcontinent = Afghanistan, Bangladesh, Bhutan, India, Maldives,† Nepal, Pakistan, Sri Lanka; East Asia = Brunei, Burma, Cambodia, China, Hong Kong,† Indonesia, Japan,† Laos, Malaysia, Mongolia,† Philippines, South Korea, Singapore,† Taiwan,† Thailand, Tibet,† Vietnam; West Africa = Benin, Burkina Faso, Cape Verde, Ghana, Guinea, Guinea-Bissau, Ivory Coast, Liberia, Mali, Mauritania, Senegal, Sierra Leone, The Gambia, Togo; East Africa = Burundi, Djibouti, Eritrea, Ethiopia, Kenya, Rwanda, Seychelles,† Somalia, Sudan, Tanzania, Uganda; Central Africa = Cameroon, Central African Republic, Chad, Congo, Democratic Republic of Congo, Equatorial Guinea, Gabon, Niger, Nigeria, São Tomé et Principe; Southern Africa = Angola; Botswana, Lesotho, Madagascar, Malawi, Mauritius,† Mozambique, Namibia, South Africa, Zambia, Zimbabwe; Central America and Carribean = Antigua and Barbuda,† Bahamas,† Barbados,† Belize, Bermuda,† Cayman Islands,† Costa Rica, Cuba,† Dominica,† Dominican Republic, El Salvador, Grenada,† Guadeloupe,† Guatemala, Honduras, Jamaica,† Haiti, Martinique,† Mexico, Netherlands Antilles,† Nicaragua, Panama, Puerto Rico,† St. Christopher and Nevis,† St. Lucia/St. Vincent,† Saint Kitts-Nevis,† The Grenadines,† Trinidad and Tobago,† Virgin Islands;† South America = Bolivia, Brazil, Colombia, Ecuador, French Guiana, Guyana, Paraguay, Peru, Suriname, Uruguay,† Venezuela.

Three quarters of all cases and 93% of the *P. falciparum* cases were seen in travelers from sub-Saharan Africa (Table 4). The crude risk for travelers to different regions varied from 1 per 100,000 travelers to Central America and the Caribbean to 357 per 100,000 in central Africa. In the multivariable analysis, OR for being diagnosed with any malaria species after return to Sweden was calculated for various risk factors (Table 1). Compared to the reference region (Central America and the Caribbean), travelers to East Africa had the highest OR for being reported with malaria, closely followed by travelers to central Africa and West Africa. The Indian subcontinent had an OR in the same “middle” range as southern Africa. Southeast Asia and South America had similar ORs, at the lower range. The malaria risk in Arab countries did not differ significantly from the risk in Central America and the Caribbean.

Malaria was significantly more often diagnosed in men than in women, as well as in the age-group <1–6 years, after adjustments were made for the various confounders. The calculated malaria risk per 100,000 travelers in 4 countries with alternative sources of travel information is shown in Table 5, with risk data based on the travel database, and the annual malaria incidence reported to WHO (*15*) as comparisons.

**Table 5:** Malaria risk for officially reported travelers compared to incidence (as reported to WHO, 2001*) and estimated rate (based on TDB) in corresponding region†

			Data from in-flight/visa		Data from TDB			
Country	Y	Cases	No. of travelers	Risk/100,000	No. of travelers	Risk/100,000	95% CI	Data from WHO incidence/100,000
Thailand	2001–2002	9	453,000	2.0	435,000	2.1	1.0–4.2	100
India	2001–2003	7	48,687	14.4	–	–	–	192
Indian subcontinent	2001–2003	25‡	–	–	72,000	35	17-71	–
Gambia	1997–2003	79	31,242	253	–	–	–	10.096
West Africa	1997–2003	242§	–	–	80,000	302	196–468	–
South Africa	1997–2001	3	98,886	3	–	–	–	61
Southern Africa	1997–2001	63¶	–	–	128,000	49	32–76	–

*Information from The Gambia was from 1999. †WHO, World Health Organization (*15*);TDB, Swedish Travel and Tourist Database; CI, confidence intervals. ‡7 cases from India, 13 cases from Afghanistan, and 5 cases from Pakistan. §6 cases from Benin, 4 cases from Burkina Faso, 31 cases from Ivory Coast, 79 cases from The Gambia, 65 cases from Ghana, 11 cases from Guinea, 5 cases from Guinea-Bissau, 11 cases from Liberia, 3 cases from Mali, 11 cases from Senegal, 11 cases from Sierra Leone, and 5 cases from Togo. ¶14 cases from Angola, 5 cases in Madagascar, 8 cases from Malawi, 13 cases from Mozambique, 3 cases from Namibia, 3 cases from South Africa, 7 cases from Zambia, and 10 cases from Zimbabwe.

## Discussion

These results are based on official reports of malaria, with data from one of the largest ongoing population-based surveys on travel patterns in Europe as denominator. The laboratory method of microscopy of a blood film for malaria is well defined, and the reporting of diagnosed malaria is believed to be relatively complete in Sweden. Since case-patients are reported both by the clinician and the laboratory, the overall sensitivity of the Swedish surveillance system is comparatively high, with >95% of diagnosed diseases being reported (*17*).

The information in the tourist and travel database and the reporting database were not fully consistent. The travel database did not contain any data on travel-related illnesses, while the official reports did not contain information on length of stay. Previous studies have shown that the duration of stay influences the risk for malaria (*5*,*12*,*13*), but this factor could not be evaluated in this study. Because the tourist and travel database classification sometimes included both malaria-endemic and malaria-nonendemic countries within the same region, some travelers who only visited regions that were not malaria-endemic were included in the denominator for the region. Therefore, the risk for the region may be underestimated, e.g., in east Asia, which includes several malaria-free countries. However, many of these countries are comparatively rare as tourist destinations.

To further evaluate the precision of the estimates from the tourist database, we also used official in-flight and visa data obtained from 4 countries. Risk estimates from the 2 sources for Thailand and The Gambia/West Africa had good agreement. For India and the Indian Subcontinent and South Africa/southern Africa, where we did not have any tourist data per country, most travelers were going to India and South Africa, respectively, while most malaria patients were from other countries in these regions. The risk estimates from the 2 sources were therefore in less agreement with each other in these regions.

We found that men had a significantly higher risk of being reported with malaria compared to women. A predominance of imported malaria infections in male patients has been documented before (*2*,*5*,*18*,*19*); men are often also less compliant with chemoprophylaxis than women (*5*). We also found that children <6 years of age had a significantly higher risk of being reported with malaria. To our knowledge, this risk has not been described before. Many of these young children belonged to immigrant families with roots in the country of infection, where they visited friends and relatives; such children are referred to as “VFRs” (*20*,*21*). Parents may be unaware of the fact that the children lack the immunity against malaria when returning “home.”

According to official statistics from the Swedish Migration Board, >775,000 persons were granted permanent residence permit in Sweden during the period 1980–2002. A large proportion of these persons came from countries where malaria is highly endemic. A survey based on Norwegian surveillance data has shown that the incidence of malaria was higher in VFRs than in people of Norwegian origin (*22*). In our study, distinguishing native Swedes from persons with an origin in other countries was not possible among controls. However, reporting data, which often includes this information, indicated that a large number of the patients were VFRs who had visited countries outside usual tourist routes, including Somalia, Ethiopia, Uganda, Bangladesh, and Pakistan. Our data thus suggest that VFRs are a risk group requiring special attention. These persons may be less inclined than other travelers to get pretravel advice and to use chemoprophylaxis against malaria (*21*,*23*).

*P. falciparum* malaria is the prime target for chemoprophylaxis to prevent death and severe disease. Although we considered doing a separate risk analysis for this species, reporting at the species level in the first year of the study was not sufficiently complete to allow for meaningful analysis. Furthermore, other malaria species also contribute substantially to malaria illness in travelers (*24*). Falciparum malaria is a clinically overt disease and will most probably be diagnosed. For other malaria species, a few cases, especially in persons with partial immunity, might be missed, thus underestimating the true risk.

No systematically collected data on chemoprophylaxis are included in this study, an obvious limitation when assessing the malaria risk. However, previously published reporting data from 2003 for *P. falciparum* infection indicate that 12 of 17 Swedish travelers had not taken any prophylaxis, and another 2 had taken drugs with insufficient effect for the country they visited. Of 34 VFRs, only a few had taken prophylaxis (*25*). Prophylaxis influences the number of cases, as do other factors associated with the behavior of individual travelers, such as use of mosquito-protective measures and the standard of housing visited, on which we do not have any information from reports. Furthermore, local malaria transmission intensity is key to the malaria risk for a traveler. Within several malaria-endemic countries, the risk for malaria varies greatly, reflecting local transmission intensity at the district level (*16*,*26*,*27*). This local variation, together with the different travel patterns within countries, may greatly influence the risk of travelers contracting malaria. For example, in Thailand, most Swedish travelers go to areas of the country in which no malaria occurs, a fact that could partly explain the low incidence in Swedish travelers compared to WHO data on local incidence. This low incidence in Swedish travelers is in line with a previously cited report on malaria risk in Danish residents traveling to Thailand (*13*), where the risk was estimated to be 2.5 per 100,000 travelers. Our corresponding figure, with denominator data from the embassy, was 2.1 per 100,000. A British study reported a slightly higher risk, 8.2 per 100,000 (*6*). All 7 Swedish travelers who contracted *P. falciparum* malaria in Thailand from 1997 to 2003 had visited regions outside of the usual charter tourist destinations, such as remote national parks and jungle areas. Another example is The Gambia, where the average Swedish tourist often stays most (or all) of the vacation in beach resorts by the coast, where the malaria transmission intensity is lower than in inland areas (*28*).

Discrepancies do exist between the calculated risks for Swedish travelers to have malaria diagnosed in Sweden after traveling to malaria-endemic regions, and the incidence rates of patients in the same regions reported to WHO. Several factors could explain these differences, including the small number of Swedish malaria cases, the fact that indigenous malaria is associated with more than just transmission intensity, i.e., poverty (*29*), duration of exposure, and different sensitivity of the surveillance and reporting systems in different countries. Data from Africa, especially, are incomplete (*29*).

In general, the order of magnitude of the relative risk for malaria in the different regions was consistent with earlier data on relative malaria risk for travelers (*9*–*12*). Based on these risk data, we divided the regions into 3 groups: sub-Saharan Africa (except for southern Africa) exhibited the greatest relative risk for malaria in returning travelers (>250), followed by India and southern Africa (relative risk [RR] ≈50) and at the lower end Southeast Asia, South America, and the Arab countries (RR <10). A number of other studies have shown higher risk for acquiring malaria in West Africa compared to East Africa (*5*,*8*). Our data show higher risk in those visiting central Africa and West Africa than East Africa, corresponding to these previous studies. Our results are representative for the overall malaria risk in Swedish travelers and are also likely to reflect the risk in travelers from other European countries.

This study confirms that the risk of a traveler’s contracting malaria is highest in Africa, south of the Sahara, and that male travelers and small children constitute groups with increased risks. Furthermore the added complexity of immigrants from malaria-endemic areas needs to be considered when discussing malaria prevention among travelers. All pretravel advice needs to be individualized for each traveler, based on the exact travel route, season, and type of travel.
